# SHIP1 phosphatase as a Late‐Onset Alzheimer’s Disease therapeutic target

**DOI:** 10.1002/alz.095377

**Published:** 2025-01-09

**Authors:** Kratika Singhal, Adam K Hamdani, Cynthia D Jesudason, Daniel E Beck, Brent Clayton, Timothy I. Richardson, Andrew D. Mesecar

**Affiliations:** ^1^ Purdue University, West Lafayette, IN USA; ^2^ Purdue, West Lafayette, IN USA; ^3^ LGenia, Fortville, IN USA; ^4^ Indiana University School of Medicine, Indianapolis, IN USA

## Abstract

**Background:**

Alzheimer’s disease (AD) is a highly complex neurological disorder, with Late‐Onset AD (LOAD) being its most common form. INPP5D has been identified as a risk gene for AD and is involved in the TREM2 signaling pathway, which is crucial for microglial activity. INPP5D encodes SHIP1, a protein phosphatase that disrupts TREM2 signaling by converting PIP3 into PIP2, thereby inhibiting the PI3K‐mediated activation of Akt‐dependent signaling, which is essential for the clearance of amyloid oligomers, fibrils, and plaques. SHIP1 is a large, multidomain protein, and many aspects of its structure and function are poorly understood.

**Method:**

We have expressed, purified, and characterized the kinetic and biophysical properties of various domain constructs of SHIP1 to better understand the roles of individual domains. Ongoing work involves screening of inhibitors using a range of biochemical and biophysical assays with different constructs of SHIP1.

**Result:**

The response of different SHIP1 domain constructs with different substrates surprisingly revealed no significant differences in kinetic parameters between different domain constructs with the same substrate suggesting that the various domains surrounding the catalytic domain do not influence catalysis in solution. However, use of a designed chemical probe with a covalent warhead that targets SHIP1 allosterically between the catalytic and C2 domains shows significant inhibition of SHIP1 (in the absence of its SH2 domain) identifying a potential druggable site. X‐ray crystallography was used to confirm the binding pose within this site. Binding affinity with additional compounds has been determined for different domain constructs using enzyme kinetics and biophysical methods including Microscale Thermophoresis (MST) and Differential Scanning Fluorescence (DSF).

**Conclusion:**

SHIP1 is highly active in vitro (solution) without much regulation of its catalytic activity by surrounding domains. A potential druggable site has been identified between the SHIP1 catalytic and C2 domains that can be targeted allosterically by small molecule compounds. These discoveries will aid in identifying new molecules that can inhibit SHIP1 as a potential therapeutic target for AD.

This research was supported by grant 1U54 AG065181 (IUSM‐Purdue TREAT‐AD Center) from the National Institute of Aging, National Institutes of Health.

